# Identifying Language Development in Children with ADHD: Differential Challenges, Interventions, and Collaborative Strategies

**DOI:** 10.3390/children11070841

**Published:** 2024-07-10

**Authors:** Dimitra V. Katsarou, Efthymia Efthymiou, Georgios A. Kougioumtzis, Maria Sofologi, Maria Theodoratou

**Affiliations:** 1 Department of Preschool Education Sciences and Educational Design, Faculty of Humanities, University of the Aegean, 85132 Rhodes, Greece; d.katsarou@aegean.gr; 2 Department of Interdisciplinary Studies, Zayed University, Abu Dhabi 144534, United Arab Emirates; 3 Department of Turkish Studies and Modern Asian Studies, Faculty of Economic and Political Sciences, National and Kapodistrian University of Athens, 15772 Athens, Greece; gkougioum@ppp.uoa.gr; 4 Laboratory of Psychology, Department of Early Childhood Education, School of Education, University of Ioannina, 45110 Ioannina, Greece; m.sofologi@uoi.gr; 5 School of Social Sciences, Hellenic Open University, 26335 Patras, Greece; m.theodoratou@nup.ac.cy; 6 Department of Psychology, School of Health Sciences, Neapolis University, 8042 Pafos, Cyprus

**Keywords:** ADHD, language development, executive function, intervention strategies, collaborative support

## Abstract

Attention Deficit Hyperactivity Disorder (ADHD) significantly influences children’s language acquisition and usage. This theoretical study explores the multifaceted impact of ADHD on language development, specifically focusing on reading and writing challenges. Existing research reveals that approximately 30% of children with ADHD show significant delays in reading proficiency. Additionally, about 40% of these children struggle with phonological processing, which directly impacts their reading and writing skills. Interventions targeting executive function training combined with phonics-based instruction have been shown to significantly improve language outcomes. This study introduces a comprehensive framework connecting these challenges to specific interventions and collaborative strategies, emphasizing the importance of a multi-disciplinary approach. This work provides perspectives on the specific connections between ADHD symptoms and language difficulties, offering detailed potential solutions based on empirical data. Moreover, it features the necessity of adopting integrated intervention strategies to advance academic outcomes and communicative competencies for children with ADHD, providing new understandings into effective educational practices.

## 1. Introduction

Attention Deficit Hyperactivity Disorder (ADHD), as outlined in the DSM-5-TR (2022), remains a prevalent neurodevelopmental disorder among children. This condition is characterized by significant difficulties in attention, hyperactivity, and impulsivity, with symptoms typically manifesting in childhood and often continuing into adulthood. Recent studies have refined the understanding of ADHD, suggesting differences in symptomatology across genders and age groups, which has important implications for diagnosis and intervention strategies [1]. Boys with ADHD tend to exhibit a more severe clinical picture, often externalizing their symptoms in the forms of hyperactivity and provocative conduct [2]. The classification of ADHD into three subtypes—combined type, predominantly inattentive type, and predominantly hyperactive–impulsive type—remains critical for diagnosis and treatment planning, highlighting the disorder’s symptom diversity [3]. The diagnosis criteria require the exhibition of six or more symptoms from either the hyperactivity–impulsivity or inattention categories for at least six months, with an onset before the age of seven [4].

This theoretical study introduces a new conceptual framework that links the multifaceted challenges faced by children with ADHD to targeted interventions, emphasizing the interconnectedness of various cognitive, linguistic, and social factors.

## 2. Attention Deficit Hyperactivity Disorder and Verbal Language Development

Research consistently indicates that a significant proportion of children diagnosed with ADHD experience delays in language development, affecting early speech onset and academic proficiency in reading and mathematics [5,6,7,8]. Existing research shows that approximately 30% of children with ADHD exhibit significant delays in reading proficiency [6,7,8], while 40% struggle with phonological processing [9,10,11,12,13,14]. These challenges are particularly pronounced in children with the combined type of ADHD [14,15]. These educational challenges are compounded by low vocabulary, trouble learning new words, a preference for simpler sentence structures, omitting sentence components, avoiding complex expressions, and a sluggish speech rate. Such challenges are persistent, reflecting the intricate obstacles these children navigate, exacerbated by core symptoms of ADHD, including distractibility and executive functioning deficits [14,16]. The prevalent distractibility among children with ADHD significantly hampers their language development, preventing effective observation and mimicking of surrounding speech patterns [14]. Phonological challenges are particularly pronounced, with children facing difficulties articulating and identifying phonemes accurately, extending to mispronunciation and rhythmic word pronunciation issues, suggesting broader auditory processing and working memory deficits [16]. Despite some progress in vocabulary acquisition, children with ADHD struggle with language application and comprehension within context, indicating a persistent barrier to effective communication and learning [17,18].

### Interventions

To address these challenges, interventions should focus on enhancing phonological awareness and vocabulary acquisition. Techniques such as phonics-based instruction, which emphasizes the relationship between letters and sounds, can improve reading skills. Speech–language therapy can also help children articulate sounds correctly and develop better speech patterns. Additionally, executive function training that targets working memory and attention can aid in reducing distractibility and improving language comprehension and application. Studies have shown that combining these interventions can result in significant improvements in reading comprehension scores [15,16].

## 3. Pragmatic Language Challenges

Pragmatic deficits represent a significant dimension of the linguistic challenges encountered by children with ADHD, closely linked to both cognitive functions and social interactions [19,20]. Impairments in executive functions, such as planning, organization, and self-regulation, notably hinder children’s ability to effectively engage in various forms of language behavior, including storytelling and discourse [21]. Research indicates that approximately 35% of children with ADHD struggle with maintaining eye contact and understanding social cues, impacting their pragmatic language skills [22]. These challenges are compounded by attentional deficits and difficulties in processing social cues, obstructing language acquisition and narrative construction capabilities [22,23,24,25]. Empirical studies, such as that of Zambrano-Sánchez et al. [26], reveal that children with ADHD display significantly lower proficiency in pragmatic and syntactic linguistic domains, emphasizing the need for targeted interventions that address these specific challenges.

### Interventions

Interventions for pragmatic language challenges should include social skills training that focuses on improving eye contact, turn-taking, and understanding social cues. Storytelling workshops can advance narrative skills, helping children organize their thoughts and communicate more effectively. Additionally, integrating executive function training with pragmatic language therapy can improve overall communication abilities. Studies have shown that children participating in these combined interventions show significant improvements in social interaction skills [26,27,28].

## 4. Comorbidity of ADHD with Specific Learning Disabilities (SLDs)

The connection of ADHD with various Specific Learning Disabilities (SLDs), including dyslexia, dysgraphia, and dyscalculia, presents complex challenges that significantly impact children’s language development and broader functional capabilities. Recent advancements in research have shed light on the high comorbidity rates between ADHD and specific learning difficulties, underscoring the nuanced challenges children face when these conditions coexist. For instance, dyslexia exhibits a notable comorbidity rate with ADHD, with estimates varying significantly across studies [28,29]. Research indicates that approximately 50% of children with both ADHD and dyslexia face compounded difficulties in reading and writing [2,3,4]. This rate is higher for children with the combined type of ADHD [5]. This complexity necessitates integrated diagnostic and therapeutic approaches to address the diverse needs of these children.

### Interventions

An integrated approach that combines reading interventions for dyslexia with strategies to manage ADHD symptoms is crucial. Multisensory instruction methods, which use visual, auditory, and kinesthetic–tactile pathways simultaneously to advance memory and learning, can be effective. Additionally, using technology-assisted tools like text-to-speech and speech-to-text software can support children in overcoming reading and writing difficulties. Studies have shown that these combined interventions can significantly improve reading fluency and comprehension [30,31,32].

## 5. Attention Deficit Hyperactivity Disorder and Written Language

Challenges in written language are closely linked to ADHD, as children with the disorder often face significant difficulties in expressing their knowledge, thoughts, perceptions, and feelings through writing. Research reveals that 45% of children with ADHD experience significant difficulties in written expression, often producing shorter and less coherent texts [33,34,35]. These difficulties are exacerbated by attention deficits, leading to challenges in following complex instructions and completing academic tasks. As a result, academic productivity is often reduced, a key indicator of school performance [36,37,38,39,40]. Children with ADHD display a lower tolerance for tasks of increased difficulty, opting to disengage and consequently producing work that is incomplete or riddled with errors, including spelling mistakes. This inconsistency in task completion time and the presence of deficient fine motor skills, along with compromised executive functions and working memory, highlight the impact of ADHD on the ability to write and complete tasks [40].

### Interventions

To support written language development, interventions should focus on improving fine motor skills and executive function capabilities. Occupational therapy can help develop the fine motor skills needed for writing. Additionally, teaching strategies like graphic organizers can help children plan and structure their writing. Breaking writing tasks into smaller, manageable steps and providing frequent breaks can also help maintain focus and productivity. Research indicates that these strategies can lead to significant improvements in the quality and coherence of written assignments [33,34,35,36,37].

## 6. Writing Challenges in Children with ADHD

Children with ADHD often display distinct writing patterns, producing simpler, shorter texts that lack the structural coherence and ideational richness found in the work of their non-ADHD peers [41,42]. These cognitive impairments directly impact children with ADHD’s writing capabilities and overall academic performance. Limited expressive language in early childhood serves as a precursor to these cognitive challenges, particularly affecting verbal comprehension and working memory by ages 7–8. This finding is especially relevant for children with ADHD, for whom these cognitive aspects are critical for successful written expression. The presence of working memory deficits in a subset of children with ADHD highlights the variability of cognitive profiles within this group and its association with academic achievement, including writing skills [41].

### Interventions

Effective interventions for writing challenges include explicit teaching of writing strategies, such as brainstorming, outlining, drafting, and revising. Providing templates and frameworks for different types of writing can help structure children’s thoughts. Regular feedback and one-on-one support can also help improve their writing skills. Utilizing technology, such as word processing programs with spell check and grammar suggestions, can assist in producing clearer and more coherent texts. Studies have demonstrated that these interventions can significantly improve writing clarity and coherence [43,44,45].

## 7. Deficits in Organizational Skills

Executive functions, encompassing cognitive processes such as working memory, flexible thinking, and self-control, play a pivotal role in effective writing. Children with ADHD often face challenges in these areas, adversely affecting their writing capabilities [5,8,13]. Targeted interventions aimed at bolstering executive functions have shown promise in enhancing the writing skills of children with ADHD. Strategies including the use of graphic organizers and breaking writing tasks into smaller steps are effective in improving their planning and organizational skills. Additionally, interventions that focus on enhancing internalized speech mechanisms, such as storytelling workshops, can improve narrative construction and coherence [46,47,48].

### Interventions

Interventions to improve organizational skills should focus on teaching children how to plan and prioritize their tasks. Using visual schedules and checklists can help them stay organized and on track. Executive function training programs that include activities to improve working memory, cognitive flexibility, and self-regulation are also beneficial. Incorporating these skills into daily routines and academic tasks can help reinforce their application. Research supports that these interventions can result in significant improvements in task completion and organization [46,47,48].

## 8. Future Research Directions

Given the complexity and broad spectrum of challenges associated with ADHD, future research should aim to explore these issues in greater depth. Large-scale, longitudinal studies are needed to understand the long-term impacts of ADHD on language development and the effectiveness of various intervention strategies over time. Additionally, research should focus on the following areas:

- Diverse populations: Studies should include a wide range of demographic groups to ensure the findings are generalizable across different cultures and socioeconomic backgrounds.
- Comorbid conditions: Further investigation into the interaction between ADHD and other learning disabilities, such as dyslexia and dysgraphia, can provide more nuanced insights into the compounded challenges these children face.
- Technological interventions: Evaluating the effectiveness of emerging technology-based interventions, such as digital storytelling and interactive learning tools, can offer new avenues for supporting children with ADHD.
- Parental and educator training: Research into the impact of training programs for parents and educators on the outcomes of children with ADHD can help optimize support strategies at home and in educational settings.

By addressing these areas, future research can build on the current understanding of ADHD and develop more effective, tailored interventions to support the language development of affected children.

## 9. Integrated Framework and Conceptualization

**Figure 1:**
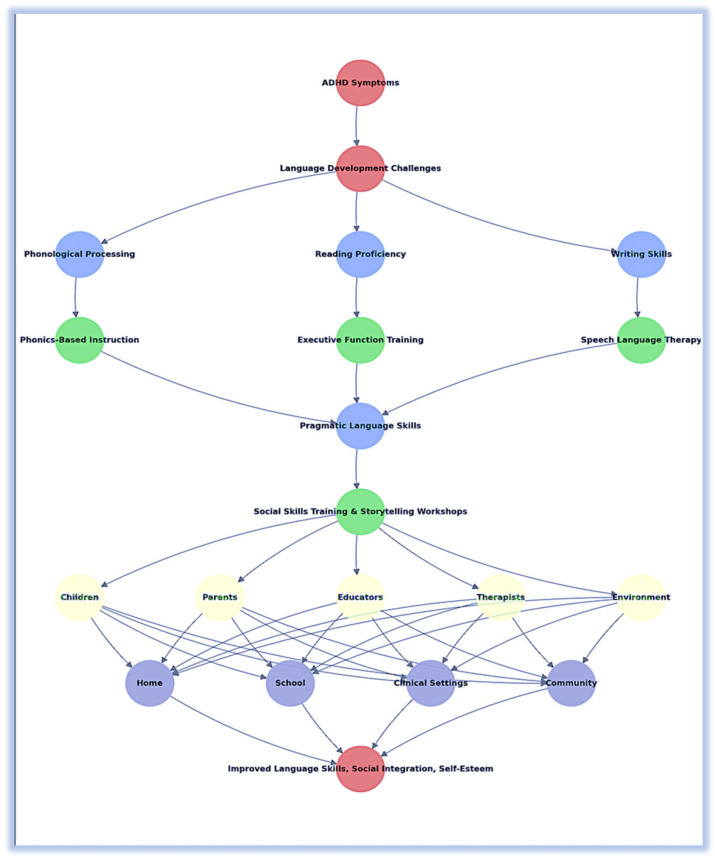
Integrated framework for addressing language development in children with ADHD.

To provide a comprehensive understanding of the multifaceted impact of ADHD on language development and propose effective intervention strategies, we have developed an integrated framework. This framework outlines the inter-relationships between ADHD symptoms, language development challenges, interventions, and the roles of different agents within various environments (Figure 1).

**Analysis:**

The integrated framework illustrates how ADHD symptoms such as inattention, hyperactivity, and impulsivity contribute to various language development challenges including phonological processing, reading proficiency, writing skills, and pragmatic language skills. Each of these challenges necessitates specific interventions:

- Phonological processing challenges are addressed through phonics-based instruction. This method emphasizes the relationship between letters and sounds, aiding in the development of decoding skills essential for reading [49].
- Reading proficiency issues are targeted with a combination of executive function training and phonics-based instruction. Executive function training enhances working memory and attention, supporting better reading comprehension [50].
- Writing skills deficiencies are improved with speech–language therapy and executive function training. These interventions help children articulate sounds correctly, develop better speech patterns, and organize their thoughts for writing [51].
- Pragmatic language skills benefit from social skills training and storytelling workshops. These activities help children improve eye contact, turn-taking, understanding social cues, and narrative construction [52].

**Roles of different agents:**

- Children: Actively engage in interventions and apply the skills learned in various contexts.
- Parents: Support interventions at home, reinforcing skills and providing a consistent environment for practice.
- Educators: Implement classroom strategies and provide individualized support to enhance the learning and application of language skills.
- Therapists: Design and oversee interventions, monitor progress, and adjust strategies as needed for effectiveness.

Environments for implementation:

- Home Environment: Parents support phonics-based instruction and reinforce social skills training through everyday interactions and structured activities.
- School Environment: Educators integrate executive function training and multisensory instruction into the curriculum, providing a supportive academic setting.
- Clinical Settings: Therapists provide targeted speech–language therapy and utilize technology-assisted tools to address specific language development needs.

**Holistic approach:**

The integrated approach addresses the multifaceted challenges of ADHD and language development by involving all agents and environments in a cohesive strategy. This confirms that interventions are effectively implemented and reinforced across different settings, leading to improved language skills, social integration, and self-esteem in children with ADHD. By linking various elements such as symptoms, challenges, interventions, and roles, the framework provides a comprehensive, structured conceptualization that highlights the importance of collaborative efforts and tailored strategies to support the holistic development of children with ADHD.

This framework accentuates the complexity of ADHD and its impact on language development, providing practical solutions to be implemented by researchers, educators, clinicians, and parents. It emphasizes the need for a coordinated, multi-disciplinary approach to effectively address the diverse needs of children with ADHD, allowing them to achieve their full communicative and academic potential.

By providing a holistic view of the inter-relationships and integrated strategies, this framework offers new insights and practical solutions for addressing the complex language development challenges faced by children with ADHD.

## 10. Conclusions

In conclusion, this theoretical study explores the complex effects of ADHD on children’s language development, covering both spoken and written communication. Drawing on the latest studies and clinical findings, ADHD significantly disrupts conventional learning and communication pathways, necessitating an integrated approach to diagnosis, intervention, and assistance. This paper delineates how ADHD affects children’s ability to develop language skills at a pace and depth comparable to their neurotypical peers. From early speech delays to complexities in narrative construction and written expression, the breadth of impact features the disorder’s profound influence on educational and communicative outcomes.

Given the multifaceted nature of ADHD, this manuscript acknowledges the challenge of covering all related issues comprehensively within its limited scope. The presence of comorbid learning disabilities, such as dyslexia and dysgraphia, further complicates the language development of children with ADHD. Notably, the exacerbation of these challenges in the presence of comorbid learning disabilities highlights the critical need for integrated diagnostic and therapeutic strategies that are sensitive to the diverse symptomatology and developmental trajectories of those affected.

Central to the discourse is the assertion that effective intervention requires a dual focus on enhancing executive functions and addressing language impairments. The discussion advocates for an integrated approach underpinned by neurodevelopmental frameworks that merges executive function training with targeted language development strategies. Such interventions exemplified by the work of leading researchers and educators hold promise for improving the academic performance, social integration, and self-esteem of children with ADHD.

Moreover, the importance of collaborative efforts involving educators, therapists, parents, and the wider community cannot be understated. A supportive environment characterized by structured, tailored, and compassionate support emerges as a cornerstone for facilitating the holistic development of children with ADHD. These efforts, while challenging, are vital in developing the potential of these children, overcoming the complexities of language acquisition with greater confidence and capability. As research continues, it is imperative that strategies for intervention and support adapt accordingly, ensuring that every child achieves their full communicative and academic potential.

## Data Availability

No new data have been reported.

## References

[1] American Psychiatric Association (2022). Diagnostic and Statistical Manual of Mental Disorders.

[2] Pennington B.F., McGrath L.M., Peterson R., Peterson R.L. (2019). Diagnosing Learning Disorders: From Science to Practice.

[3] Boada R., Willcutt E.G., Pennington B.F. (2012). Understanding the comorbidity between dyslexia and attention-deficit/hyperactivity disorder. Top. Lang. Disord..

[4] Cortese S., Kelly C., Chabernaud C., Proal E., Di Martino A., Milham M.P., Castellanos F.X. (2012). Toward systems neuroscience of ADHD: A meta-analysis of 55 fMRI studies. Am. J. Psychiatry.

[5] Barkley R.A. (2014). Attention-Deficit Hyperactivity Disorder: A Handbook for Diagnosis and Treatment.

[6] Rocco I., Corso B., Bonati M., Minicuci N. (2021). Time of onset and/or diagnosis of ADHD in European children: A systematic review. BMC Psychiatry.

[7] Soto E.F., Irwin L.N., Chan E.S., Spiegel J.A., Kofler M.J. (2021). Executive functions and writing skills in children with and without ADHD. Neuropsychology.

[8] Chen Y., Tsao F.M., Liu H.M., Huang Y.J. (2022). Distinctive patterns of language and executive functions contributing to reading development in Chinese-speaking children with ADHD. Read. Writ..

[9] Jepsen I.B., Hougaard E., Matthiesen S.T., Lambek R. (2022). A systematic review and meta-analysis of narrative language abilities in children with Attention-Deficit/Hyperactivity Disorder. Res. Child Adolesc. Psychopathol..

[10] Carruthers S., Taylor L., Sadiq H., Tripp G. (2022). The profile of pragmatic language impairments in children with ADHD: A systematic review. Dev. Psychopathol..

[11] Matte-Landry A., Boivin M., Tanguay-Garneau L., Mimeau C., Brendgen M., Vitaro F., Dionne G. (2020). Children with persistent versus transient early language delay: Language, academic, and psychosocial outcomes in elementary school. J. Speech Lang. Hear. Res..

[12] Daucourt M.C., Erbeli F., Little C.W., Haughbrook R., Hart S.A. (2020). A Meta-Analytical Review of the Genetic and Environmental Correlations between Reading and Attention-Deficit/Hyperactivity Disorder Symptoms and Reading and Math. Sci. Stud. Read..

[13] Sciberras E., Mulraney M., Silva D., Coghill D. (2017). Prenatal risk factors and the etiology of ADHD—Review of existing evidence. Curr. Psychiatry Rep..

[14] Gooch D., Thompson P., Nash H.M., Snowling M.J., Hulme C. (2016). The development of executive function and language skills in the early school years. J. Child Psychol. Psychiatry.

[15] Rapport M.D., Eckrich S.J., Calub C., Friedman L.M. (2020). Executive function training for children with attention-deficit/hyperactivity disorder. The Clinical Guide to Assessment and Treatment of Childhood Learning and Attention Problems.

[16] Parsons L.Q., Cordier R., Munro N., Joosten A., Speyer R. (2017). A systematic review of pragmatic language interventions for children with autism spectrum disorder. PLoS ONE.

[17] Redmond S.M. (2016). Language impairment in the attention-deficit/hyperactivity disorder context. J. Speech Lang. Hear. Res..

[18] Delage H., Frauenfelder U.H. (2020). Relationship between working memory and complex syntax in children with Developmental Language Disorder. J. Child Lang..

[19] Hanna C.H.F. (2023). Phonic Faces as a Method for Improving Decoding for Children with Persistent Decoding Deficits. Ph.D. Thesis.

[20] Kapnoula E.C., Jevtović M., Magnuson J.S. (2024). Spoken Word Recognition: A Focus on Plasticity. Annu. Rev. Linguist..

[21] Katsarou D. (2023). Developmental Language Disorders in Childhood and Adolescence.

[22] El Sady S.R., Nabeih A.A., Mostafa E.M., Sadek A.A. (2013). Language impairment in attention deficit hyperactivity disorder in preschool children. Egypt. J. Med. Hum. Genet..

[23] Kessler P.B., Ikuta T. (2023). Pragmatic Deficits in Attention Deficit/Hyperactivity Disorder: Systematic Review and Meta-Analysis. J. Atten. Disord..

[24] American Speech-Language-Hearing Association (ASHA) (2024). Spoken Language Disorders. https://www.asha.org/practice-portal/clinical-topics/spoken-language-disorders/.

[25] Kyriacou M., Köder F. (2023). Exploring the pragmatic competence of adults with ADHD: An eye-tracking reading study on the processing of irony. OSF Regist..

[26] Zambrano-Sánchez E., Cortéz J.A.M., del Río Carlos Y., Moreno M.D., Cortés N.A.S., Hernández J.V., Cervantes T.E.R. (2023). Linguistic alterations in children with and without ADHD by clinical subtype evaluated with the BLOC-S-R test. Investig. Discapac..

[27] Abdalla E.A.F., Hassan S.M., Boshnaq M.H. (2023). Assessment of Pragmatic Aspect of Language in Children Diagnosed with Attention Deficit Hyperactivity Disorder. QJM Int. J. Med..

[28] Aksoy Ş.G. (2024). The Comorbidity of Specific Learning Disorders in Attention Deficit Hyperactivity Disorder. Compr. Med..

[29] Wang J., Hu E., Ansari D., Gaab N. (2024). Does familial risk of dyscalculia or/and dyslexia impact brain activity during a numerical magnitude comparison task in kindergarteners?. OSF Regist..

[30] Maleki S., Hassanzadeh S., Rostami R., Pourkarimi J. (2024). Design and effectiveness of a reading skills advancement program based on executive functions specifically for students with comorbid dyslexia and ADHD. Psychol. Sci..

[31] Calderoni S., Coghill D. (2024). Advancements and challenges in autism and other neurodevelopmental disorders. Front. Child Adolesc. Psychiatry.

[32] Mohl B., Ofen N., Jones L.L., Robin A.L., Rosenberg D.R., Diwadkar V.A. (2015). Neural dysfunction in ADHD with Reading Disability during a word rhyming Continuous Performance Task. Brain Cogn..

[33] Tahıllıoğlu A., Bilaç Ö., Erbaş S., Barankoğlu Sevin İ., Aydınlıoğlu H.M., Ercan E.S. (2024). The association between cognitive disengagement syndrome and specific learning disorder in children and adolescents with ADHD. Appl. Neuropsychol. Child..

[34] Papaeliou F.X. (2012). The Development of Language: Theoretical Approaches and Research Evidence from Typical and Deviant Language Behavior.

[35] Protopapas A., Protopapas A. (2012). The Development of Speech Perception. Language Development and Disorders.

[36] Willcutt E.G., Pennington B.F. (2000). Comorbidity of reading disability and attention-deficit/hyperactivity disorder: Differences by gender and subtype. J. Learn. Disabil..

[37] Malegiannaki A., Messinis L., Papathanasopoulos P. (2012). Clinical Child Neuropsycholog.

[38] Miller D.J., Komanapalli H., Dunn D.W. (2024). Comorbidity of attention deficit hyperactivity disorder in a patient with epilepsy: Staring down the challenge of inattention versus nonconvulsive seizures. Epilepsy Behav. Rep..

[39] Louick R., Muenks K. (2022). Leveraging motivation theory for research and practice with students with learning disabilities. Theory Pract..

[40] Sarid M., Lipka O., Bar-Kochva I. (2024). Adults with learning difficulties in post-secondary education. Front. Psychol..

[41] Patil S., Apare R., Borhade R., Mahalle P. (2024). Intelligent approaches for early prediction of learning disabilities in children using learning patterns: A survey and discussion. J. Auton. Intell..

[42] Rollins S.P. (2020). Teaching Vulnerable Learners: Strategies for Students Who Are Bored, Distracted, Discouraged, or Likely to Drop Out.

[43] Marinopoulou M. (2023). The Relationship between Cognition and ESSENCE in Childhood. Ph.D. Thesis.

[44] Young N.D., Bonanno-Sotiropoulos K., Citro T. (2018). Paving the Pathway for Educational Success: Effective Classroom Strategies for Students with Learning Disabilities.

[45] Flood J., Lapp D., Brice Heath S. (2015). Handbook of Research on Teaching Literacy through the Communicative and Visual Arts.

[46] Otero T.M., Barker L.A., Naglieri J.A. (2014). Executive function treatment and intervention in schools. Appl. Neuropsychol. Child.

[47] Mason L., Brady S. (2021). Promoting Executive Functions during Writing Process.

[48] Martinussen R., Mackenzie G. (2015). Reading comprehension in adolescents with ADHD: Exploring the poor comprehender profile and individual differences in vocabulary and executive functions. Res. Dev. Disabil..

[49] Torgesen J.K. (2002). The prevention of reading difficulties. J. School Psychol..

[50] Diamond A., Ling D.S. (2016). Conclusions about interventions, programs, and approaches for improving executive functions that appear justified and those that, despite much hype, do not. Dev. Cogn. Neurosci..

[51] Berninger V.W., O’Malley M.J. (2011). Evidence-based diagnosis and treatment for specific learning disabilities involving impairments in written and/or oral language. J. Learn. Disabil..

[52] Adams C., Lockton E., Freed J., Gaile J., Earl G., McBean K., Nash M., Green J., Vail A., Law J. (2012). The Social Communication Intervention Project: A randomized controlled trial of the effectiveness of speech and language therapy for school-age children who have pragmatic and social communication problems with or without autism spectrum disorder. Int. J. Lang. Commun. Disord..

